# Non-game like training benefits spoken foreign-language processing in children with dyslexia

**DOI:** 10.3389/fnhum.2023.1122886

**Published:** 2023-03-10

**Authors:** Katja Junttila, Anna-Riikka Smolander, Reima Karhila, Mikko Kurimo, Sari Ylinen

**Affiliations:** ^1^Cognitive Brain Research Unit, Department of Psychology and Logopedics, Faculty of Medicine, University of Helsinki, Helsinki, Finland; ^2^Logopedics, Welfare Sciences, Faculty of Social Sciences, Tampere University, Tampere, Finland; ^3^Department of Signal Processing and Acoustics, Aalto University, Espoo, Finland

**Keywords:** digital game-based learning, gaming, foreign-language learning, automatic speech recognition, dyslexia

## Abstract

Children with dyslexia often face difficulties in learning foreign languages, which is reflected as weaker neural activation. However, digital language-learning applications could support learning-induced plastic changes in the brain. Here we aimed to investigate whether plastic changes occur in children with dyslexia more readily after targeted training with a digital language-learning game or similar training without game-like elements. We used auditory event-related potentials (ERPs), specifically, the mismatch negativity (MMN), to study learning-induced changes in the brain responses. Participants were 24 school-aged Finnish-speaking children with dyslexia and 24 age-matched typically reading control children. They trained English speech sounds and words with “Say it again, kid!” (SIAK) language-learning game for 5 weeks between ERP measurements. During the game, the players explored game boards and produced English words aloud to score stars as feedback from an automatic speech recognizer. To compare the effectiveness of the training type (game vs. non-game), we embedded in the game some non-game levels stripped of all game-like elements. In the dyslexia group, the non-game training increased the MMN amplitude more than the game training, whereas in the control group the game training increased the MMN response more than the non-game training. In the dyslexia group, the MMN increase with the non-game training correlated with phonological awareness: the children with poorer phonological awareness showed a larger increase in the MMN response. Improved neural processing of foreign speech sounds as indicated by the MMN increase suggests that targeted training with a simple application could alleviate some spoken foreign-language learning difficulties that are related to phonological processing in children with dyslexia.

## 1. Introduction

Developmental dyslexia is manifested as reading and writing impairment despite sufficient opportunity for instruction (Bishop and Snowling, 2004). The prevalence of dyslexia ranges from 5 to 20%, depending on definition (Wagner et al., 2020). In addition to reading and writing difficulties, dyslexia is often associated with difficulties in verbal short-term memory (Szenkovits and Ramus, 2005), rapid automatized naming (Bexkens et al., 2015), and phonological processing (Ramus et al., 2013). The difficulties in phonological processing may also be linked to impaired auditory processing of speech (Gu and Bi, 2020), yet the findings have been controversial in this respect. Nevertheless, children with dyslexia typically have difficulties learning spoken novel words (Adlof et al., 2021) and they struggle especially with phonologically demanding tasks which require detecting subtle differences between spoken words (Alt et al., 2017). Consequently, dyslexia does not only impair reading and writing in the native language, but it often hampers learning foreign languages (Downey et al., 2000; Helland and Kaasa, 2005; Soroli et al., 2010; Ylinen et al., 2019).

In our previous study (Ylinen et al., 2019), we found a link between native-language literacy skills and foreign-language word representations in the brain, suggesting that typical readers may benefit from classroom teaching and exposure more than dyslexic readers. A recent study found a hybrid technology game useful for children with dyslexia for foreign language receptive vocabulary learning (Eden and Shmila, 2022). The hybrid technology used by Eden and Shmila (2022) focused on spelling and translation. However, it remains unexplored whether more targeted training techniques focusing on listening to and producing speech could support spoken foreign-language learning in dyslexia (see Jarsve and Tsagari, 2022) and, in addition, which features of targeted training are particularly beneficial for them. Since children with dyslexia have reading difficulties, speech-based training can be expected to be more suitable for their foreign-language learning than reading-based training. Nevertheless, it is not clear which other features of targeted foreign-language training are particularly beneficial for children with dyslexia because studies on foreign-language learning interventions are scarce. For example, our earlier study shows that game-based learning supports the learning of foreign speech sounds more than the use of a non-game application in typically developing children (Junttila et al., 2022), raising the question whether this holds also for children with dyslexia. Digital language-learning games can enhance player’s motivation to learn by being easy to use and providing reward and feedback, right amount of challenge, control and autonomy, clearly defined goals, and interactivity (Acquah and Katz, 2020). Therefore, they could be an attractive way for children with dyslexia to learn foreign languages. However, it is not known whether digital language-learning games induce plastic changes in the brain and support the establishment of long-term memory representations similarly for children with dyslexia as for typically reading children. Therefore, this study aimed to determine whether targeted game-based training supports foreign-language learning in dyslexia. Specifically, we compared the effectiveness of playing a digital language-learning game and rehearsing in a non-game application in children with dyslexia.

The game we used focuses on spoken foreign-language learning and it is based on listening and producing speech (Karhila et al., 2017; Ylinen and Kurimo, 2017; Ylinen et al., 2021; Junttila et al., 2022). In the game, players hear English words and produce them aloud, which activates the connections between temporal and frontal language areas that have been found to be deficient in people with dyslexia (Boets et al., 2013; Wang et al., 2017; see Vandermosten et al., 2012 for a review). Speech training with auditory-motor activation might strengthen these connections needed in accessing phonological representations (Temple et al., 2003). Importantly, however, we also expect that auditory-motor activity associated with the speech training interacts with brain activity associated with gaming elements that have been suggested to enhance learning-induced plastic changes in the brain (Junttila et al., 2022; see also Oei and Patterson, 2013; Nahum and Bavelier, 2020, for game effects beyond language).

Producing words aloud in the game enables an automatic speech recognizer (Karhila et al., 2017, 2019) to assess the utterances and to give on-line feedback: player’s performance is rewarded with one to five stars as a function of its accuracy. Players need to collect the stars to proceed in the game. This is expected to activate the reward system of the brain and to motivate players to produce foreign speech as accurately as possible. Together, these processes could typically result in the efficient establishment of accurate brain representations for foreign speech sounds and words. However, some dyslexic readers have been found to show atypical structure or function of striatum (Krishnan et al., 2016), which may affect the ability to learn from the game. Since striatum is the core area of the reward system of the brain, atypical function of striatum may hinder the processing of feedback that the game provides to reinforce learning. This may diminish the benefits of feedback and the game-based approach for learning. Therefore, we hypothesized that if the processing of feedback in striatum is indeed atypical in dyslexia (Krishnan et al., 2016), getting feedback from the game could benefit typical readers more than dyslexic readers and result in weaker game-based training effects in dyslexia.

To assess the effects of targeted training with the game and the non-game, in particular, long-term memory representations established in the brain, we used auditory event-related potential (ERP) measurements. Specifically, we focused on the mismatch negativity (MMN), an ERP component which is elicited when a stimulus violates a previously formed prediction (Winkler, 2007). The MMN can be elicited by presenting a regular sound stream consisting of so-called standard sounds and occasional deviant sounds (oddball paradigm). Its peak is typically observed 100–250 ms after the deviance onset and it is largest at fronto-central electrode sites when referenced to the average of mastoids (Sams et al., 1985). The MMN is considered particularly feasible for the present study for two reasons. Firstly, in addition to acoustic deviance the amplitude of MMN is enhanced by long-term memory traces for speech sounds (Näätänen et al., 1997) or individual spoken words (Pulvermüller et al., 2001). Processing ability reflected by the MMN governs attentive discrimination (Tiitinen et al., 1994; Amenedo and Escera, 2000), and previous training studies (Tremblay et al., 1998; Ylinen et al., 2010; Tamminen et al., 2015; Junttila et al., 2022) show that the MMN is feasible to tap the establishment of brain representations for non-native speech sounds and words that are in the focus of the present study. Therefore, measuring the MMN response allowed us to evaluate plastic changes in brain due to behavioral foreign-language training. Secondly, the elicitation of MMN does not require attention (Näätänen et al., 1978; Fitzgerald and Todd, 2020), and thus it is a very useful tool for studying the target group of this study, namely, children who may have short attention span or who may not be motivated to perform tasks (Ylinen et al., 2021). Previous MMN studies have also robustly shown differences in speech processing between individuals with dyslexia and their controls (e.g., Schulte-Körne et al., 1998; Bruder et al., 2011; van Zuijen et al., 2013).

## 2. Materials and methods

### 2.1. Ethics

The study was conducted according to the Declaration of Helsinki and it was approved by the University of Helsinki Ethical Review Board in the Humanities and Social and Behavioral Sciences. Participation was voluntary. A written informed consent was acquired from the participant’s guardians and an oral informed consent from the participants. The compensation for participation was one cinema ticket per hour of testing and EEG measurement.

### 2.2. Participants

The study included two groups of participants: children with dyslexia (*N* = 24) and fluently reading control children (*N* = 24). The control children are a subsample of the participants in a previous study (Junttila et al., 2022). The control children were selected so that they matched the children in dyslexia group by age and sex. According to parental reports, the participants were 7–11 years old monolingual native speakers of Finnish. The dyslexia group included 14 girls and 10 boys (mean age 9 years 8 months, SD 12 months). Correspondingly, the control group consisted of 14 girls and 10 boys (mean age 9 years 8 months, SD 10 months).

The participants were screened with pre-tests that measured literacy, cognitive skills, and phonological skills (see details below) to verify that they met the inclusion criteria and to decide the allocation to the two groups. The inclusion criteria for all participants of the study were as follows: age of 7–11 years, being a Finnish-speaking monolingual with no head injuries, having normal hearing, normal vision or vision corrected to normal with eyeglasses, and obtaining a minimum of 6 standard points in the four Wechsler Intelligent Scale for Children IV (WISC-IV; Wechsler, 2010) subtests used in the study (Block Design, Digit Span, Coding, and Vocabulary). There were also additional criteria that were different for the dyslexia group and the control group. To be included in the dyslexia group, the participants had to meet the following criteria: a maximum of 6 standard points in the Finnish reading and writing skill test LukiLasse (Häyrinen et al., 1999) word reading task and either a maximum of 6 standard points in the LukiLasse dictation task or a maximum of level score 3/9 in another Finnish literacy test task, Ala-asteen lukutesti (ALLU; Lindeman, 1998) word segmentation task. The additional inclusion criteria for the control group were a minimum of 7 standard points in the LukiLasse word reading and dictation tasks and a minimum of level score 4 in the ALLU word segmentation task.

### 2.3. Cognitive pre-tests

Participants’ knowledge of the target words used as stimuli during the EEG experiment was tested before participating in the first EEG measurement. Each stimulus word and pseudoword was presented *via* headphones one by one. After presenting each word, the children were asked whether the heard items sounded familiar to them. They were also asked whether they knew the meanings of the words that sounded familiar. The same task was conducted after training to indicate learning to recognize the words and learning the meanings of the words.

Participants’ reading, writing, cognitive, and phonological skills were assessed with standardized neuropsychological tests in Finnish. Writing skills were assessed with LukiLasse dictation task where participants are required to write dictated words and sentences without time limit. Reading skills were assessed with LukiLasse word reading task and ALLU word segmentation task. LukiLasse word reading task is used to assess reading accuracy and speed. It requires reading aloud a list of words within a time limit of 2 min. In a similar vein, ALLU word segmentation task assesses technical reading skills. In this task, children read strings of letters without spaces and segment the strings into words by drawing vertical lines between the word boundaries within a time limit of 3.5 min. The test has two standardized versions (A and B) with a different set of word strings. The children did the version A of the ALLU test during the pre-test session before the training period and the version B after the training period right before the final EEG recording. In addition to literacy skills, other cognitive skills that may affect performance in our tasks were measured to ensure that the two groups did not differ in this respect. Perceptual reasoning skills were assessed with block design subtest from the Wechsler WISC-IV, auditory short-term memory was assessed with WISC-IV forward and backward digit span, eye hand coordination and processing speed were assessed with WISC-IV coding, and vocabulary was assessed with WISC-IV vocabulary.

In addition, since dyslexia is typically characterized by slow naming and poor phonological awareness, Rapid Automatized Naming Test (RAN, Ahonen et al., 2003) subtests colors and letters were used to assess naming skills and Common Unit test was used to assess phonological awareness. In Common Unit test the child is presented with spoken pseudoword pairs *via* headphones. The task is to name the common phoneme in the two words. First, there is a practice phase where three practice pairs are presented. The experimenter corrects the wrong answers and tells what the right one is in the practise phase. After that, there are 15 trials where each pair is presented once. The length of the pseudowords vary between 5 and 12 Finnish phonemes. Each correct answer scores one point and no feedback is given during the test trials.

The test performance was compared between the groups with independent samples *t*-tests (Table 1). No differences between the groups were found in WISC-IV block design, WISC-IV vocabulary, WISC-IV coding, and RAN colors. In addition to tests used for group allocation (LukiLasse word reading, LukiLasse dictation, ALLU word segmentation), groups also differed in WISC-IV digit span, RAN letters, and Common Unit task, where the control group performed better than the children with dyslexia. These differences were expected because impairments in these skills are typical in dyslexia (Wagner and Torgesen, 1987; Vellutino et al., 2004; Peters et al., 2020; Andreola et al., 2021).

**TABLE 1:** Participants’ literacy, cognitive, and phonological skill scores.

Test	Children with dyslexia: standard score/skill level/raw score (±SD)	Controls: standard score/skill level/raw score (±SD)	*t*-test
LukiLasse word reading	3.29 (±1.83)	10.13 (±2.42)	*t* = −11.04, *p* = <0.001
LukiLasse dictation	5.13 (±3.44)	10.54 (±1.35)	*t* = −7.18, *p* = <0.001
ALLU word segmentation	1.88 (±0.68)	4.71 (±1.04)	*t* = −11.16, *p* = <0.001
WISC-IV block design	10.17 (±3.32)	10.42 (±2.81)	*t* = −0.28, *p* = 0.780
WISC-IV digit span	8.46 (±1.53)	10.58 (±3.27)	*t* = −2.88, *p* = 0.006
WISC-IV vocabulary	10.58 (±2.47)	10.79 (±1.61)	*t* = −0.35, *p* = 0.731
WISC-IV coding	10.13 (±3.01)	10.75 (±3.04)	*t* = −0.72, *p* = 0.478
RAN colors	53.96 (±10.25)	49.79 (±13.89)	*t* = 1.18, *p* = 0.243
RAN letters	38.13 (±9.17)	30.42 (±5.18)	*t* = 3.59, *p* = <0.001
Common unit	4.33 (±3.32)	9.92 (±2.69)	*t* = −6.41, *p* = <0.001

Group means are given as standard scores for LukiLasse and WISC-IV, skill level for ALLU, and raw scores for RAN (time in seconds) and common unit.

### 2.4. EEG experiment

#### 2.4.1. EEG stimuli

Similarly as in our previous gaming study (Junttila et al., 2022), the stimuli presented during the EEG recordings were spoken English words and pseudowords with voiceless and voiced dental fricative phonemes /θ/ and /ð/, respectively. These phonemes were chosen because they are not part of the Finnish phonological system, and we wished to ensure that the stimulus words contain phonemes foreign to Finnish speakers to study learning effects. Although the children may have been exposed to these phonemes before the experiments, they had not learned to pronounce or distinguish the phonemes. The choice of the phonemes is also supported by the fact that syllables containing /θ/ as a foreign phoneme have previously been shown to elicit an MMN response in a similar age group (Jost et al., 2015). In addition, we aimed to choose words that would be unfamiliar so that the participants would not know their meanings in advance. Therefore, we checked English textbooks commonly used in schools to choose words that were likely not included in their vocabulary. This way we ended up choosing the words *healthy* [’helθi] and *feather* [’feðə] and formed corresponding pseudowords from them by replacing the target fricatives with stops (resulting in healty* [’helti]* and feder* [’fedə]*). The stimuli were recorded in a sound-attenuated studio. A list of English words and their minimal-pair counterpart pseudowords were given to a native English speaker who pronounced them several times. The best exemplars of the words *healthy* and *feather* and pseudowords healty* and feder* were chosen from the recordings to be used as stimuli.

**FIGURE 1:**
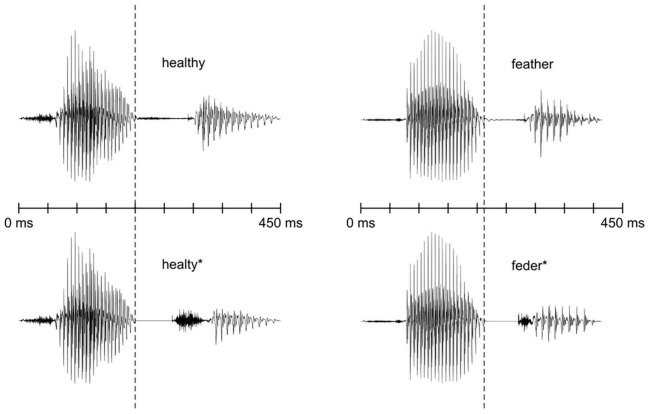
Waveforms of the stimulus sounds. The time points where the pseudowords deviate from their word counterparts are marked with dashed lines.

The recorded words and pseudowords were modified using Praat software Version 5.1.45 (Boersma and Weenink, 2010). To make sure that the word and the pseudoword could not be distinguished before the target phonemes, we used beginnings of the words *healthy* and *feather* as the beginnings of the pseudowords (Figure 1). The word *healthy* was cross-spliced at zero-crossing at 200 ms and the first part of the word was used as the beginning of the pseudoword healty*. Similarly, the word *feather* was cross-spliced at zero-crossing at 220 ms and the first part was used as the beginning of the pseudoword feder*. The pitch contours of *healthy* and *feather* were adopted to healty* and feder*, respectively.

As the aim of the study was to compare within-subject effects of the game training and the non-game training regardless of the foreign phoneme type, the responses to both *feather* and *healthy* were combined for analysis purposes.

#### 2.4.2. EEG recordings

**FIGURE 2:**
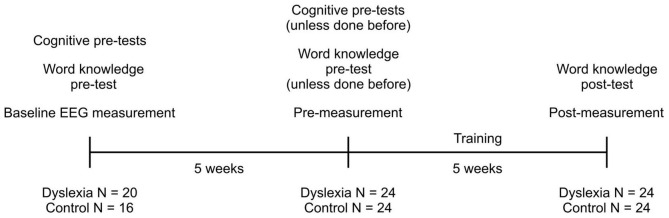
Timeline of the tests, EEG measurements, and training.

EEG was measured on two or three occasions, namely, a baseline measurement, a pre-training measurement, and a post-training measurement. All children participated in the pre-training measurement and the post-training measurement, whereas only part of the children participated in the baseline measurement (20 of the children in the dyslexia group and 16 of the children in the control group). The baseline measurement took place prior to the pre-training measurement, and it was conducted to assess and control for the effects of repeated EEG measurements and out-of-game exposure to English (nevertheless, inability to participate in three measurements was not considered an exclusion criterion). The time between the baseline measurement and the pre-measurement, as well as between the pre-measurement and the post-measurement was 5 weeks on average (Figure 2). The participants played the language learning game containing both the game levels and the non-game levels for training the target phonemes between the pre-measurement and the post-measurement.

EEG was recorded with BioSemi ActiveTwo system (BioSemi Inc., Amsterdam, and The Netherlands) and with Biosemi ActiView Version 7.07 EEG acquisition software using a 64-channel cap. Additional electrodes were placed on the tip of the nose and the mastoids for re-referencing, near the outer canthi of the eyes for horizontal eye movements, and under the left eye for vertical eye movements. The measurements were conducted in a sound and electrically attenuated room, where the participants sat on a comfortable chair. The participants chose a film to watch during the measurement. The film was muted but the subtitles were shown in Finnish. Headphones were used to present the auditory stimuli binaurally. The volume was set at a comfortable hearing level of 60 dB. The participants were instructed to ignore the stimuli and to concentrate on the film instead.

The stimuli were presented in four different sequences: two oddball sequences and two standard-only sequences. The oddball sequences had a repeating standard pseudoword that was occasionally replaced by a deviant word. A standard pseudoword feder* (*p* = 0.8) and a deviant word *feather* (*p* = 0.2) were used in one oddball sequence focusing on /ð/ and a standard pseudoword healty* (*p* = 0.8) and a deviant word *healthy* (*p* = 0.2) were used in the other one focusing on /θ/. In both oddball sequences, the standard pseudoword was presented 480 times and the deviant word was presented 120 times. Standard-only sequences had the real words from the oddball sequences presented 180 times as repeating standards. The word *feather* (*p* = 1) was presented in one standard-only sequence and the word *healthy* (*p* = 1) in the other. All sequences had an inter-stimulus interval (ISI) of 500 ms. Presentation^®^ software (Version 17.2, Neurobehavioral Systems, Inc., Berkeley, CA)^1^ was used to present the stimulus sequences. The four sequences were presented in a pseudorandom order for each participant and measurement session so that the presentation order of sequences containing words trained with the game and words trained with the non-game were counterbalanced. Each EEG measurement session lasted 2 h, including preparation.

#### 2.4.3. EEG data processing

The EEG data processing was conducted with BESA Research 7.0 software (BESA GmbH, Gräfelfing, Germany). Bad EEG channels were interpolated using the data from other channels. To improve the signal-to-noise ratio, the data were re-referenced to the average of left and right mastoids. Children’s EEG data are typically noisier than those of adults. Thus, in line with our previous work (Junttila et al., 2022), a band pass filter of 1.5–20 Hz with a slope of 24 dB/octave was used to remove slow drifts from the data that are beyond the MMN range and could distort the MMN amplitude quantification (Picton et al., 2000; Sinkkonen and Tervaniemi, 2000; Nenonen et al., 2005; Kalyakin et al., 2007; Jost et al., 2015). BESA Research Artifact Correction was used to detect and correct the horizontal and vertical eye movements. The data were then divided into epochs using a −100–800 ms time window relative to the stimulus onset. The 5 first epochs of each sequence were rejected, as were all epochs contaminated by artifacts exceeding ± 75 μV. Average waveforms were created for each stimulus type separately using the accepted epochs. Responses to the stimuli derived from standard-only blocks were subtracted from the responses to identical stimuli in deviant positions to form difference waveforms, and the baselines were corrected at the −100–0 ms pre-stimulus interval.

Grand average waveforms for the game condition and the non-game condition were formed for both groups separately. To compare the effects of the game and the non-game, the ERP waveforms for the game condition and the non-game condition were constructed by combining the responses of both target words. The MMN response is typically most prominent on fronto-central regions in data referenced to mastoids, and thus nine electrodes (F3, Fz, F4, FC3, FCz, FC4, C3, Cz, and C4) from that area were selected for analysis. The peak latency of the MMN response was determined from a grand-average waveform over both groups, both conditions, all time points, and all nine selected electrodes. The mean MMN amplitudes for both groups, both conditions, all time points, and all electrodes were then quantified from individual difference waveforms (deviant minus standard-only, i.e., the responses to the same stimulus) using a ± 20 ms time window centered at this average peak latency of 334 ms.

### 2.5. Training

**FIGURE 3:**
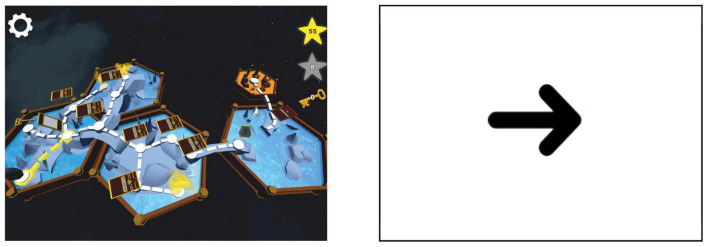
An example of a game level **(left)** and a non-game level **(right)**.

Between the last two EEG measurement times, the participants played “Say it again, kid” (SIAK) that is a language-learning game targeting spoken English and running on Windows laptops or Android tablets. The players used microphone headsets for playing. In the SIAK game, the players can move around on a game board and whenever they encounter a card, the card pops up and introduces an English word produced by a native English speaker (Figure 3, left). The card shows a picture associated with the word and the players hear the word spoken in Finnish to ensure they understand the meaning of the word. Then they hear the word spoken in English. The players’ task is then to say the word aloud in English, trying to imitate the model pronunciation as closely as they can. The players’ utterances are recorded and played back to them along with the original English pronunciation. An automatic speech recognition (ASR) technology, which is optimized for children’s voices (Karhila et al., 2017, 2019), evaluates the players’ utterances and, as feedback, awards one to five stars based on how close it was to the model pronunciation. The total number of stars the player has scored is shown on the top right-hand corner of the game screen (Figure 3, left). Scoring more stars opens new paths on the game boards. The game has visually different levels, and each level ends with a test card, where players can test their learning. The test card shows a picture of one word learned during the level but does not play a model pronunciation. The players need to produce the correct word and score at least three stars to unlock a next level. If the players at first do not succeed, they can replay the card containing the test word, practice pronouncing the word as many times as they like, and then go back to the test card to try to score enough stars.

The children played the game on average 16.3 min (control 16.8 min, dyslexic 15.7 min) per play session. They played on average 1.1 play sessions (control 1.0, dyslexic 1.1) a day on 2.8 days (control 2.7, dyslexic 3.0) a week during a period of 4.3 weeks (control 4.4, dyslexic 4.2). Altogether, the children with dyslexia played the game 209 min (SD = 42.9, range 149–323 min) and the control children played the game 197 min (SD = 48.9, range 145–341 min). All children completed all levels of the game. An independent samples *t*-test was conducted to compare the playing times between the control group and the dyslexia group. No significant difference was found [*t*(46) = 0.87, *p* = 0.39, 95% CI = −15.17, 38.26].

The SIAK game had altogether 27 levels, yet only six of them contained words with the target phonemes /θ/ (e.g., *healthy, mouth, three*) and /ð/ (e.g., *feather, mother, this*). Out of these six levels, three were normal game levels with the target sounds and three were non-game levels with the target sounds. The non-game levels had English word imitation tasks for the target sounds similarly to the game levels but did not have any game-like elements (Figure 3, right). In the non-game levels, the players were not free to explore and choose the order of the items like in the game levels, but rather the presentation order of the words was forced. Instead of the colorful game board of the game levels, the non-game levels had a black arrow on a white background. Also, the players did not score stars or receive other feedback in the non-game levels. Otherwise, the non-game training was similar to the game training: Players heard spoken words in Finnish and English and they needed to imitate the English ones. The English stimuli were counterbalanced across the level type: Half of the control group and half of the dyslexia group trained words containing the phoneme /θ/ with the game levels and words containing /ð/ with the non-game levels. The other halves of the control and dyslexia groups trained words containing the phoneme /ð/ with the game levels and words containing /θ/ with the non-game levels.

There were about 15 different words to learn at each level of the game. The players could freely explore the game levels and decide how many times they practised each word (with the exception of the non-game levels where the order and number of words were fixed). The number of times words were practised with the game levels and with the non-game levels was controlled within participants. To ensure equal amount of practice of the words containing the target phonemes at the game levels (levels 16, 20, and 25) and the non-game levels (levels 17, 21, and 26), the free-to-explore game levels with the target sounds were always presented before the non-game levels with the other target sounds. The number of times players practiced words with the target phonemes and EEG stimulus words at the game levels was recorded. During the following non-game level, words with the other target phonemes as well as the EEG stimulus words were presented a matching number of times. Thus, each player got equal amount of training consisting of listening to and producing speech with the game and the non-game.

Word learning during training, as indicated by familiarity with the word form and knowledge of word meaning, was tested after the training period just before the EEG post-measurement similarly as before the first EEG measurement in both groups. In addition, to assess the learning of sound production for two target phonemes /θ/ and /ð/ by children with dyslexia, we evaluated their utterances collected by SIAK. The utterances were evaluated using the automatic speech recognition algorithm (Karhila et al., 2019) used in the game. We counted the relative number of utterances containing the correct /θ/ and /ð/ phonemes out of all attempts for the first session and the last session containing these phonemes. The learning was defined as a change between the relative numbers of correct utterances.

### 2.6. Statistical analyses

Differences in the learning of words, trained with the game or with the non-game (i.e., the same words as those used as EEG stimuli), were examined with McNemar’s tests. Two different types of learning were assessed: learning to recognize the word form (familiarity) and learning the meaning of the word. The tests were conducted for both groups separately. In addition, we used paired one-tailed *t*-tests to examine the learning to produce the target phonemes correctly in the dyslexia group.

Differences in MMN amplitude change between the two groups after training with the game or the non-game (post-test minus pre-test) were compared with a linear mixed model analysis. The analysis was conducted with IBM SPSS Statistics, Version 27 software. The analysis included Group (dyslexic – fluent), Treatment (game – non-game), Anteriority, and Laterality as factors and the change in MMN amplitude between the baseline measurement and pre-training measurement as a covariate. A separate paired-samples *t*-test was conducted to compare difference between baseline and pre-test within the dyslexia group’s game condition.

To study the connections between neural training effects and literacy, RAN, and phonological awareness, we performed correlation analyses (Pearson’s r). To this end, the MMN amplitude change after training with the game or the non-game was averaged across the nine fronto-central electrodes. Literacy score was calculated by taking the average of the z-scores of LukiLasse word reading task, LukiLasse dictation task, and ALLU word segmentation task. RAN score was the average of RAN speed z-scores of color and letter tasks. In both composite scores, negative values indicate below-average and positive values above-average performance. Benjamini-Hochberg correction (Benjamini and Hochberg, 1995) was used to adjust for multiple comparisons. False discovery rate (FDR) was set at 0.05.

## 3. Results

Both EEG stimulus words sounded familiar to one child in the control group and four children in the dyslexia group before the first EEG recording session. In addition, 11 children in the control group and 8 children in the dyslexia group found one of the words familiar sounding. None of the children in either group knew what the words meant. After the training period, the stimulus words learned with the game sounded familiar to 20 children in the control group and 19 children in the dyslexia group. The stimulus words learned with the non-game sounded familiar to 17 children in the control group and 22 children in the dyslexia group. No familiarity difference between the words learned with the game or the non-game was found in either group according to the McNemar’s test (control: *p* = 0.453, dyslexia: *p* = 0.453). After training with the game, 12 children in the control group and 9 children in the dyslexia group had learned the meanings of the words, whereas after training with the non-game, 9 children in the control group and 5 children in the dyslexia group had learned the meanings. According to the McNemar’s test, no difference between the meanings learned with the game and the non-game was, however, found in either group (control: *p* = 0.549, dyslexia: *p* = 0.219). For speech production, *t*-tests revealed a difference for the children with dyslexia in learning to produce the correct phonemes with the game and the non-game [*t*(22) = 1.78, *p* = 0.044, 95% CI = −0.01, 0.16]. The learning was on average 7.5 percentage points stronger in the non-game situation than in the game situation.

**FIGURE 4:**
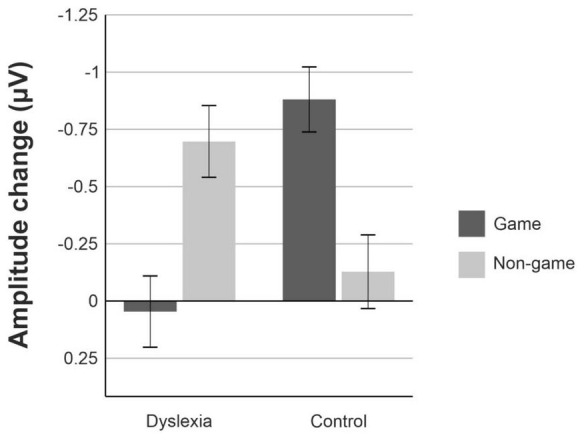
The mean amplitude changes between the pre-training and post-training measurements for the dyslexia group and the control group. Amplitude changes are averaged over the nine electrodes selected for analysis. Error bars represent the standard error of the mean.

**FIGURE 5:**
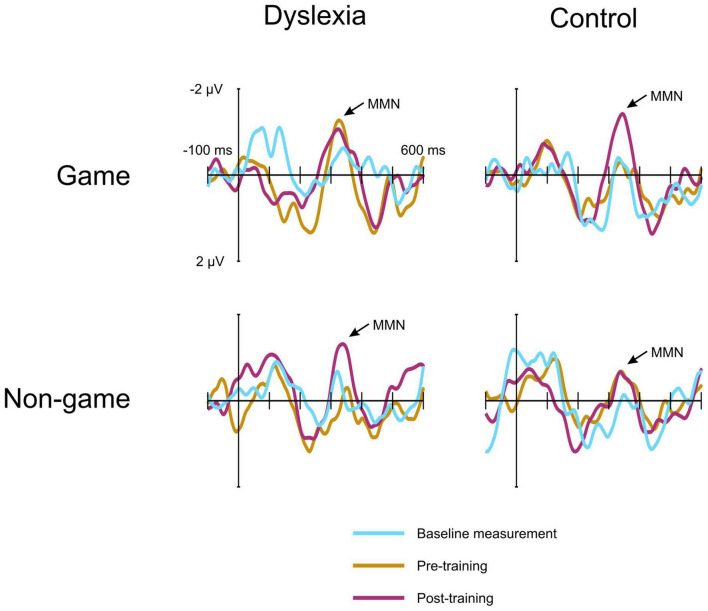
The MMN responses of the dyslexia group and the control group at electrode FCz. The responses are shown at the baseline measurement, pre-training, and post-training. Negativity is plotted up. The game and the non-game responses are measured from the same children in each group.

**FIGURE 6:**
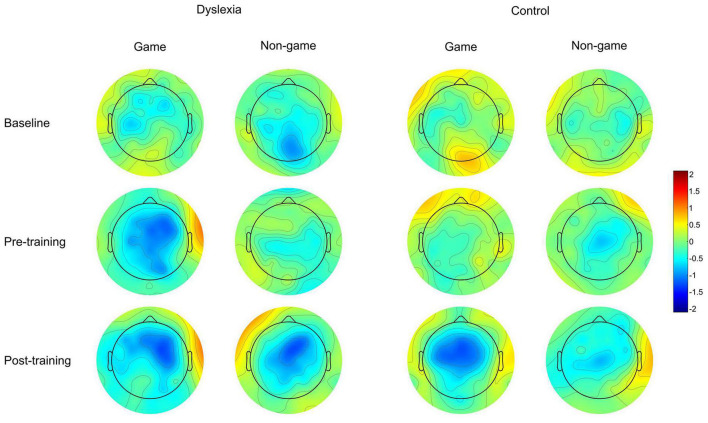
Scalp topography maps for the MMN response of the dyslexia group and the control group. The scalp topographies are shown at the baseline measurement, pre-training, and post-training. Amplitude is shown in μV.

For the MMN data, the linear mixed model revealed a significant interaction of Group and Treatment, [F(1, 550.53) = 17.19, *p* = <0.001] (Figures 4, 5). The estimated marginal means were evaluated at the average MMN amplitude change between the baseline measurement and pre-measurement (−0.31 μV) (Table 2). Pairwise comparison showed that in the dyslexic reader group, training with the non-game increased the MMN amplitude more (−0.66 μV) than training with the game (−0.07 μV) (*p* = 0.001, 95% CI = 0.23, 0.95) (Figures 5, 6). In the control group, training with the game increased the MMN amplitude more (−0.88 μV) than training with the non-game (−0.38 μV) (*p* = 0.013, 95% CI = 0.10, 0.89). No significant difference was found between baseline and pre-test within the dyslexia group’s game condition [*t*(19) = 1.48, *p* = 0.156, 95% CI = −0.24, 1.42].

**TABLE 2:** Estimated marginal means (EMM) of the MMN amplitude changes between the pre-measurement and the post-measurement in μV for the dyslexia group and the control group for the game and non-game conditions.

				95% CI	
**Group**	**Condition**	**EMM**	**S.E.**	**Lower**	**Upper**
Dyslexia	Game	−0.07	0.32	−0.70	0.57
	Non-game	−0.66	0.32	−1.30	−0.01
Control	Game	−0.88	0.36	−1.61	−0.15
	Non-game	−0.38	0.36	−1.12	0.35

The estimates are evaluated at the average MMN amplitude change between the baseline measurement and the pre-measurement.

**FIGURE 7:**
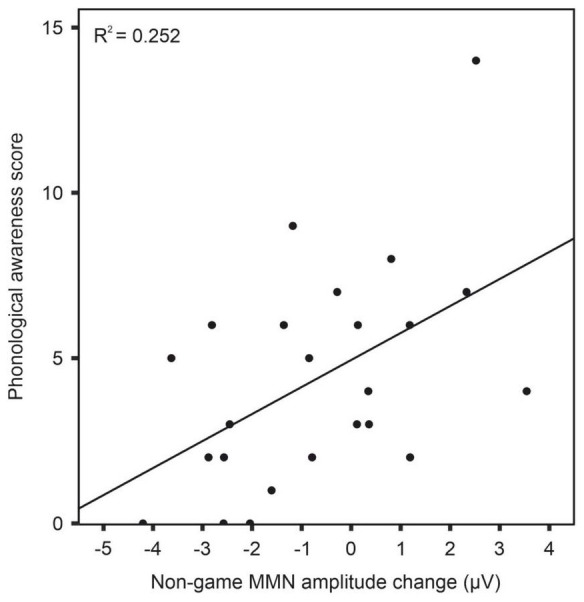
Correlation between phonological awareness and MMN amplitude change after training with the non-game in dyslexia group. Note that the MMN amplitude change reflects the difference between post-test and pre-test.

Correlation analyses revealed a significant correlation between phonological awareness and MMN amplitude change after training with the non-game in the dyslexia group [r(22) = 0.50, *p* = 0.012]. A larger MMN amplitude increase (more negative) was associated with poorer phonological awareness (Figure 7 and Table 3). No other statistically significant correlations were found (Tables 3–6).

**TABLE 3:** Correlations in the dyslexia group between the test performance and MMN amplitude change after training with the non-game.

Test	*r*	Raw *p*-value	Rank	Critical value at 0.05
Phonological awareness	0.50	0.012	1	0.017
Literacy	0.17	0.439	2	0.033
RAN	−0.16	0.462	3	0.050

**TABLE 4:** Correlations in the control group between the test performance and MMN amplitude change after training with the non-game.

Test	*r*	Raw *p*-value	Rank	Critical value at 0.05
RAN	0.16	0.445	1	0.017
Phonological awareness	−0.16	0.448	2	0.033
Literacy	−0.02	0.917	3	0.050

**TABLE 5:** Correlations in the dyslexia group between the test performance and MMN amplitude change after training with the game.

Test	*r*	Raw *p*-value	Rank	Critical value at 0.05
RAN	−0.24	0.260	1	0.017
Literacy	0.13	0.544	2	0.033
Phonological awareness	0.09	0.686	3	0.050

**TABLE 6:** Correlations in the control group between the test performance and MMN amplitude change after training with the game.

Test	*r*	Raw *p*-value	Rank	Critical value at 0.05
Literacy	0.47	0.022	1	0.017
Phonological awareness	0.38	0.068	2	0.033
RAN	−0.17	0.430	3	0.050

## 4. Discussion

This study aimed to investigate whether playing a digital language-learning game or training with a non-game would benefit the foreign-language learning of children with dyslexia and induce more plastic changes in the brain as indicated by MMN responses. We compared the change in MMN amplitude before and after training with the game and the non-game in children with dyslexia and typically reading control children. In addition, we examined whether articulatory or phonological learning with the game or the non-game is connected with literacy, rapid naming, and phonological awareness, which are typically impaired in dyslexia. In the dyslexia group, training with the non-game increased the MMN responses more than training with the game. By contrast, in the control group, training with the game was more effective than training with the non-game. In the dyslexia group, the MMN amplitude increased more after training with the non-game in those children who had poorer phonological awareness. The children with dyslexia also learned to produce the target phonemes better with the non-game than with the game.

The children with dyslexia showed larger change in the MMN responses after training with the non-game than with the game and thus appeared to benefit more from training with the non-game. The control group showed an opposite effect, benefiting more from training with the game. Because this finding is based on an interaction effect between Group and Treatment, it cannot be explained, for example, by acoustic properties of the stimuli. The pattern of results cannot be explained by other stimulus properties either, because the words and phonemes learned with the game and with the non-game were counterbalanced, so that half of the children within each group learned the target word *healthy* with the game and the target word *feather* with the non-game and vice versa. The ERP waveforms for the game and non-game conditions were created for each group separately by combining the responses to *healthy* and *feather* trained with the game and the responses to *healthy* and *feather* trained with the non-game, respectively. Thus, differences in word or phoneme difficulty or frequency cannot account for the effect. Further, each child trained words both with the game and with the non-game. Therefore, learning differences between the game and the non-game were compared in the same children within the dyslexia group, and likewise within the control group. Accordingly, training effects between the game and the non-game condition within the groups cannot be explained by individual differences between children training with the game and the non-game. Note also that the interaction between Group and Treatment was significant after controlling for the effects of repeated EEG measurements or other exposure with a baseline measurement that was used as a covariate in statistical tests. It is, therefore, unlikely that our training effects reflected in the interaction were explained by spontaneous learning during the EEG measurements or constant exposure to English outside the training (e.g., in classroom, *via* media). The different gains in the MMN amplitude in dyslexia group and the control group are, therefore, best explained by the type of training, that is, whether the training of the target sounds was conducted with the game or the non-game.

Although we have tried to control for factors that could affect our training effects, the data pattern may, nevertheless, rise some questions. Looking at MMN waveforms and scalp topographies, one may be puzzled by the difference between baseline and pre-test MMNs in the dyslexia gaming condition: compared to the baseline, pre-test MMN seems enhanced. To determine the consistency of this difference, we separately compared these conditions within this group with a paired-samples *t*-test. The difference was not significant, suggesting that it was not consistent. Perhaps the fact that not all children participated in the baseline measurement accounts for some variation in the amplitude. One may also ask whether watching subtitled films during the MMN measurements may have affected the participant groups differently. Using subtitled films is a standard procedure in MMN studies, including those addressing spoken words (Pulvermüller and Shtyrov, 2006). No effect of subtitles on MMN has been found during speech stimulation (Pettigrew et al., 2004) and subtitled films have been used also in children with dyslexia (Widmann et al., 2012). We cannot fully rule out the possibility that subtitles could affect the two groups differently, although even if this was the case, the effects are more likely to affect obligatory responses than the MMN (Pettigrew et al., 2004). However, it is very unlikely that the use of subtitles accounts for the current effect since our results are based on an interaction rather than a group main effect. The gaming effect cannot be directly caused by the subtitles because the gaming manipulation was present only in training and not in the MMN measurement. Any stimulus effects interacting with the subtitles are ruled out by counterbalancing the stimuli across training types. The means by which subtitles could potentially modify the interaction indirectly is by affecting children’s attention differently, if that had an effect on MMN. However, a thorough review by Sussman (2007) states that although attention may affect MMN elicitation through task requirements and regularity formation as part of auditory scene analysis, simply attending to deviants does not alter the amplitude of the MMN component. This suggests that attention effects do not explain the interaction. Therefore, we argue that the interaction is due to activation of brain representations that has been differently influenced by the training type in the two groups.

The difference between the current game and the non-game lies in feedback, freedom of choice, and rich visual features in the former, as opposed none of them in the latter. All these features may activate the reward system of the brain (see Marco-Pallarés et al., 2007; Wittmann et al., 2007; Leotti and Delgado, 2011; Murayama et al., 2015). In the game, the players gained stars as feedback based on how well they imitated the model word. In the non-game, the players did not get any feedback. Differences in the function of the reward system of the brain may explain why children with dyslexia benefited more from the non-game training, whereas the control children benefited more from the game training. In typical learners, feedback during speech and speech-sound learning activates striatal structures and this activation contributes to successful learning (Yi et al., 2016; Feng et al., 2019). Children in the control group benefited more from playing the game than training with the non-game, which is likely due to the activation of the reward system in the brain. Since it has previously been argued that dyslexia is characterized by atypicality in the striatum (Krishnan et al., 2016), in the dyslexia group the inefficiency of striatal functioning may result in poorer learning of words and speech-sounds in the game. Thus, it is plausible that the dyslexia group did not benefit from the feedback, freedom of choice, or novel visual features provided by the game the way the control group did.

Unlike the non-game, the game condition included colorful graphics in form of a game board with multiple choices of what to explore next. These features may increase, for example, children’s level of arousal, interest, engagement, and attention (Park and Lim, 2007; Oei and Patterson, 2013). These processes have likely enabled gaming elements to enhance the learning of the control children, which is in line with previous studies on gaming benefits for children’s foreign-language learning (Liu and Chu, 2010; Aghlara and Tamjid, 2011; Franciosi, 2017; Tsai and Tsai, 2018). A previous study found benefits of hybrid technology use on foreign language receptive vocabulary learning (translation and writing) for children with dyslexia (Eden and Shmila, 2022). That study compared a hybrid technology intervention to a traditional technology intervention, where both interventions had similar visual elements and provided feedback to the learners. In the present study, however, the children with dyslexia benefited more from the non-game training, which was stripped of all the game-like elements and was thus visually much simpler. Furthermore, no feedback was given in the non-game training. Because of these differences in the study design, the findings of the present study are not in contradiction with the findings of Eden and Shmila (2022). Rather, the differences might shed light on why the non-game training was more effective for children with dyslexia. Since children with reading difficulties have been found to also have difficulties in inhibiting unattended visual information (Facoetti et al., 2003), the lack of rich visual elements may have been one factor contributing to the finding that the children with dyslexia benefited more from the non-game training (however, see Figure 7 for individual variation within the dyslexia group in the non-game). Thus, at least in those individuals with more learning benefit from the non-game than from the game, attention may have been drawn to other elements in the gaming view than to just the current card and the word to learn. Another possibility may lie in the individual differences in optimal arousal level for learning (Hallam et al., 2002). The arousal level induced by the game may have been too high for some individuals with dyslexia, although for some others it may have been optimal. However, it is somewhat puzzling that earlier gaming studies have not shown such effects in children with dyslexia; rather, gaming has been shown to produce significant learning gains, for example, in word reading (Ronimus et al., 2019; Carvalhais et al., 2020). Perhaps then, gaming elements do not draw attention from the exposure or processes that are most critical for performing the task (e.g., here encoding of the phonological units is critical for their production, which is the main task). In contrast, the gaming elements could diminish the allocation of attention to exposure or processes that support learning but are not critical for the task at that particular moment. For example, if players wanted to proceed quickly on the game board in the current setup, they may have not focused on monitoring their speech or listening to their own utterances that were played to them after they had uttered each word, and they also may have not compared them to the model pronunciations that were also re-played. Thus, the game features may have distracted speech monitoring and self-assessment of one’s utterances if they urged the child to proceed quickly in the game.

In the dyslexia group, learning with the non-game was correlated with phonological awareness. The children with poorer phonological awareness showed a larger increase in the MMN amplitude, which may reflect alleviation of their phonological processing difficulties. The non-game training thus benefited more those children who had weaker phonological skills to start with. The training, based on listening to and producing foreign language speech without possibly distracting game elements, may have specifically tapped phonological skills, which has been found to benefit children with dyslexia (Snowling and Hulme, 2011). The connection between poor phonological awareness and learning with the non-game suggests that this kind of training may be especially helpful for the children with inferior phonological skills. No correlation was, however, found between the MMN amplitude increase and literacy scores, although dyslexia has been shown to impair learning foreign languages (Downey et al., 2000; Helland and Kaasa, 2005; Soroli et al., 2010; Ylinen et al., 2019). A possible explanation could be that the training period in this study was too short and literacy improvements in dyslexia require longer-term learning.

This study compared children with dyslexia and control children that were matched by age and sex. As children with dyslexia have poorer reading skills than the control children, comparing them with control children with matching reading-skill or phonological-skill level regardless of age could provide additional information. Therefore, further study is needed to investigate whether there are differences in the effectiveness of digital game-based foreign-language learning between children with dyslexia and reading-skill or phonological-skill matched control children.

Although significant learning effects were observed in brain responses, no significant differences between training with the game or the non-game were found in word familiarity or learning the meanings of the words in either group of children. As discussed in our previous paper (Junttila et al., 2022), brain responses may more strongly reflect foreign speech-sound learning, whereas word familiarity and learning the meanings may reflect word learning. Nevertheless, word familiarity results point to the same direction as the brain-response data: the dyslexia group tended to improve more in the non-game, whereas the controls tended to improve more in the game. In a similar vein, these and brain-response results are supported by the dyslexic children’s speech production data, showing significantly larger improvement for the speech sounds trained in the non-game than in the game. The finding of similar patterns in MMN, speech production, and perhaps also word familiarity, but a different pattern with word meanings, supports our earlier interpretation that the learning effects induced by our paradigm concern primarily phonology and speech-sound processing rather than word meanings.

The results of this study provide information that can be used for developing foreign-language teaching practices and designing digital foreign-language learning applications. Whereas typically reading children benefit from game-based learning, training with an application that has a relatively simple layout and less possibly distracting elements may be more useful for children with dyslexia – at least those with poorest phonological skills. As neural level individual differences can have an important role in language learning (Turker et al., 2021); it is noteworthy that we also saw large individual differences within the groups. Although at the group level we found a gain in the dyslexia group’s MMN amplitude in response to training with the non-game, in some individuals the MMN amplitude decreased. It has been hypothesized that dopamine-related genes are associates with language-learning differences (Wong et al., 2012), which could shed light why elements activating the reward system support the learning of some individuals but not others. Therefore, offering different kinds of teaching and learning approaches and personalizing language learning could aid a wider group of learners.

Note that although in the present setup our non-game training increased the brain responses more than the game training in children with dyslexia, the current data does not, however, suggest that gaming does not work as a learning technique in dyslexia. For example, gaming could motivate children with dyslexia to rehearse useful skills longer than a non-game application, and this kind of long-term practice could bring the children the intended benefits, whereas a non-game application that is eventually not used at all would not. The present results also do not tease apart the effects of different game features. Thus, the differences found here in learning with the game and the non-game may be related to one or two features of the game or a combination of all features, and their effect may also be individual. Further investigation of training techniques optimal in dyslexia is, therefore, needed. Nevertheless, we may speculate that visually simple design might be beneficial for many children with dyslexia.

To conclude, the results show that unlike typically reading children, children with dyslexia may benefit more from visually simple training than from visually rich game training. In the dyslexia group, the simple non-game training enhanced the MMN amplitude more than the game training. The non-game training increase in the dyslexia group was connected to phonological awareness evaluated before the training: the MMN increase was larger in the children with poorer phonological awareness. This could indicate that articulatory training with a simple training application could remediate some spoken foreign-language learning difficulties related to phonological processing deficits.

## Data availability statement

The raw data supporting the conclusions of this article will be made available by the authors, without undue reservation.

## Ethics statement

The studies involving human participants were reviewed and approved by the University of Helsinki Ethical Review Board in the Humanities and Social and Behavioral Sciences. Written informed consent to participate in this study was provided by the participants’ legal guardian/next of kin.

## Author contributions

KJ: conceptualization, methodology, formal analysis, investigation, data curation, writing — original draft, visualization, and funding acquisition. A-RS: software, investigation, and data curation. RK: methodology and software. MK: methodology and funding acquisition. SY: conceptualization, methodology, writing — review and editing, supervision, project administration, and funding acquisition. All authors approved the submitted manuscript.

## Footnotes

1
www.neurobs.com
